# Prevalence and Types of Coinfections in Sleeping Sickness Patients in Kenya (2000/2009)

**DOI:** 10.1155/2011/248914

**Published:** 2011-09-11

**Authors:** J. M. Kagira, N. Maina, J. Njenga, S. M. Karanja, S. M. Karori, J. M. Ngotho

**Affiliations:** ^1^Department of Tropical Infectious Diseases, Institute of Primate Research, P.O. Box 24481, Nairobi 00502, Kenya; ^2^Department of Biochemistry, Jomo Kenyatta University of Agriculture and Technology, P.O. Box 62000, Nairobi 00200, Kenya; ^3^National Sleeping Sickness Referral Hospital, KARI-TRC Alupe, P.O. Box 399, Busia, Kenya; ^4^Department of Biochemistry and Molecular Biology, Egerton University, P.O. Box 536, Egerton 20115, Kenya

## Abstract

The occurrence of coinfections in human African trypanosomiasis (HAT) patients was investigated using a retrospective data of hospital records at the National Sleeping Sickness Referral Hospital in Alupe, Kenya. A total of 31 patients, 19 males and 12 females, were diagnosed with HAT between the years 2000 and 2009. The observed co-infections included malaria (100%), helminthosis (64.5%), typhoid (22.5%), urinary tract infections (16.1%), HIV (12.9%), and tuberculosis (3.2%). The species of helminthes observed included *Ancylostoma duodenale* (38.7%), *Ascaris lumbricoides* (45.7%), *Strongyloides stercoralis* (9.7%), and *Taenia* spp. (3.2%). The patients were also infected with *Entamoeba* spp. (32.3%) and *Trichomonas hominis* (22.6%) protozoan parasites. The main clinical signs observed at the point of admission included headache (74.2%), fever (48.4%), sleep disorders (45.2%), and general body pain (41.9%). The HAT patients were treated with suramin (early stage, 9/31) and melarsoprol (late stage, 22/31). In conclusion, the study has shown that HAT patients have multiple co-infections which may influence the disease pathogenesis and complicate management of HAT.

## 1. Introduction

Human African trypanosomiasis (HAT) is endemic in sub-Saharan Africa, and at least 60 million people are at risk [1]. In Kenya, the disease is caused by *Trypanosoma brucei rhodesiense* and occurs in the western part of the country. Prior to 1990, most HAT cases originated from Lambwe Valley in Nyanza Province, whereas, from 1990 to 2009, the majority of cases came from new focus in Busia, Teso, and Bungoma districts in Western Province [2]. The aggressive control of the tsetse fly vector by government and donor-funded agencies and anthropogenic activities have caused a significant reduction in the number of HAT cases in Kenya, such that, between 2000 and 2009, only 31 cases were reported (Hospital data, Alupe subdistrict hospital, Kenya). However, it should be noted that, for *T.b. rhodesiense*, there is widespread underreporting due to poverty, misdiagnosis, and mode of surveillance used [3].

HAT occurs in areas which are also endemic of other tropical diseases, the main one being malaria. This is because these diseases are vector transmitted, and the vectors (tsetse flies and mosquitoes) share the same habitat. Despite a wide overlap between these diseases, published reports of coinfections are scarce in the literature. In East Africa, *T.b. rhodesiense* is characterized by a wide spectrum of clinical signs [4]. For first-stage infections, the main clinical signs and symptoms are not specific and include fever, headache, body pains, loss of appetite, and enlarged lymph nodes. The presence of a chancre at the site of tsetse bite may be indicative for a trypanosome infection. Symptoms of second-stage infections mainly include neuropsychiatric symptoms and motor problems. In a recent study, 79.7% of the HAT patients from Tanzania and 2.9% from Uganda had malaria [4]. The investigators also showed that malaria and HIV coinfections did not have any significant implication on the clinical presentation and treatment outcomes of *T.b. rhodesiense* infections. A similar study has not been undertaken in Kenya.

In sub-Saharan African countries, hospitals managing HAT do not have a significant mandate to tackle other tropical diseases. In most cases, there is no policy as regards the management of concurrent infections. This has also been attributed to lack of accurate data on these coinfections [5]. Further, Rhodesian form of HAT is zoonotic, and it is not clear cut as to whether the disease should be researched and controlled by veterinary or medical authorities [5]. For hospitals which have been earmarked to undertake research and management of a single disease such HAT, recent decline in the number of cases has reduced the workload of the staff, and this may lead to downgrading the disease from “neglected” to simply being ignored [6]. Thus, it would be important for these hospitals to handle multiple diseases. Due to lack of accurate data on occurrence of these coinfections, their importance is not known. The current study was aimed at establishing the infections which are associated with HAT in Kenya. Further, the paper reviews literature of HAT coinfections and their possible implication on the pathogenesis and management of respective diseases.

## 2. Materials and Methods

Retrospective data from National Sleeping Sickness Referral Hospital (NSSRH) in Alupe, Kenya, were used in this study. The patients are normally referred to the hospital by other health centres or can report directly to the hospital once they have signs associated with the disease. The hospital records of the HAT patients were also examined for data on sex, age, area of origin, parasites/coinfections, and disease stage. The patients are normally screened for HAT and other tropical diseases including malaria, typhoid, and helminthiasis using standard parasitological methods. For HAT, the patients underwent routine diagnosis as described by WHO [7]. Briefly, blood obtained from the patients was first checked for the presence of trypanosomes using the direct wet smear and capillary centrifugation technique methods. For trypanosome negative specimens, the blood was injected to Swiss white mice which were monitored for parasitemia development. Disease stage determination was by examination of cerebrospinal fluid (CSF) using the WHO criteria [7] in which patients with trypanosomes in the CSF and/or a cell count >5 cells/mm^3^ were classified as late stage. The number of CSF white cell counts was determined by counting the cells using the Neubauer hemocytometer method.

Other diseases were diagnosed as described by Cheesebrough [8]. Malaria diagnosis was based on the identification of plasmodium on a thin blood film and/or on a thick blood film stained with Giemsa [8]. Typhoid was diagnosed using the Widal method [8]. Stool was also collected from the patients and analysed for the presence of helminth eggs and protozoa ova using wet smears stained with iodine. Blood from patients with signs which could be attributed to human immunodeficiency virus (HIV) was examined using ELISA kits. Tuberculosis was diagnosed by sputum microscopy and chest radiography. 

Data was entered into Ms Excel, and descriptive statistics were prepared in terms of tables and graphs. Further, the data was exported to Ms Staview statistical package and analysed to determine the correlations between the occurrence of HAT and other diseases.

## 3. Results

A total of 31 patients, 19 males and 12 females, were diagnosed with HAT between 2000 and 2009. The 31 HAT cases were reported in 2000 (12), 2001 (7), 2002 (10), 2006 (1), and 2009 (1). Two cases (6.4%) which had co-infection with HIV resulted in death, while the rest were successfully treated. The mean age of the patients was 32.5 ± 2.3 (range = 14–57), with the majority (72.4%) being less than 40 years old. The patients which were in early and late stage were 9 (29%) and 22 (70%), respectively. The average period of stay at the hospital was 40 (range = 13–63) days and was not significantly (*P* > 0.05) associated with disease stage. Females were admitted for a slightly longer time than males (41 versus 38 days), but the difference was not significant (*P* = 0.44). The HAT drugs were provided for free by World Health Organization (WHO). The average cost for other treatment requirements (including cost for hospital bed, food, and all ailments except HAT) was Ksh 800 (11.4 USD) per day. This amounted to Ksh 32,000 (457 USD) for the average 40 days the patient spent at the hospital, and this was paid by the Government of Kenya. 

The number of coinfections is indicated in Table 1. In descending order the most common parasitic diseases included malaria, helminthiasis, amoebiasis, and trichomoniasis. The commonest helminthes included *Ascaris lumbricoides*, *Ancylostoma duodenale*, *Strongyloides stercoralis*, and *Taenia* spp. Other coinfections included typhoid, urinary tract infection (UTI), human immunodeficiency virus-acquired immune deficiency syndrome (HIV-AIDS) and tuberculosis. The most common coinfections consisted of patients (29%) with a combination of with HAT, malaria, helminth, and gastrointestinal protozoa (Table 2). Twenty-six percent (26%) of the patients had combination of HAT and malaria (Table 2). 

Sex, age, and disease stage had no significant effects (*P* > 0.05) on types of coinfections found in the patients.

### 3.1. Clinical Signs Observed at the Point of Admission

The mean haemoglobin (Hb) levels at admission and at discharge were 10.9 (SEM = 0.3) and 12 g/dL (SEM = 0.5), respectively. This showed that there was a significant (*P* < 0.05) improvement on Hb levels after treatment.  Table 3 shows the clinical signs observed in patients at the point of admission. In descending order, the main clinical signs observed in the patients included headache (74.2%), fever (48.4%), sleep disorders (45.2%), and general body pain (41.9%). The clinical signs which could be associated with central nervous system (CNS) involvement were observed in 21 patients (67.7%) and included sleep disorders (insomnia and/or day time sleep), stupor, paralysis, convulsions, and coma. There were significantly (*P* < 0.05) more late-stage (86.3%) than early-stage (22.2%) patients with CNS signs. 

### 3.2. Drugs Administered to the Patients

Since the HAT cases were due to *T.b. rhodesiense*, early-stage disease was treated using 5 injections of suramin at a dosage of 20 mg/kg body weight (a maximum of 1 g/injection) at intervals of 5–7 days, while late-stage disease was treated using Melarsoprol (3.6 mg/kg repeated every 7 days for 4 weeks). The main type of drugs which were used to manage the coinfections included trypanocidals, antimalarials, analgelsics, antiinflammatory, antibiotics, dewormers, antihistamines, and antiamoebas. The mean number of drug types given to the patients was 5.5 (range = 3–9), with 74% of the patients receiving 5 or more drug types.

## 4. Discussion

The current study reports the occurrence of 31 HAT cases and the associated coinfections between 2000 and 2009. It is important to note that most of the HAT cases were reported between 2000 and 2002, with only 2 cases occurring between 2006 and 2010. Further, there is wide underreporting (69%) [9] of HAT cases due to *T.b. rhodesiense*, and, thus, there could be other cases which were not addressed by the referral hospital. The mean hospital stay per patient in the current study was 40 days which is lower than that of neighbouring Uganda (47 days) [1]. The treatment costs (hospitalization and other non-HAT drugs) recorded in this study were higher than those reported for other HAT studies (120–147 USD per patient) [1, 7]. The costs in our study were higher because of the high bed charges and occurrence of coinfections which required multiple tests and subsequent treatment drugs. However, it is imperative to note that the costs for the HAT patients (average of 457 USD) were catered for by the Kenyan government—which can be compared to the 6.5 USD per capita available for healthcare in Kenya [10]. There were more male than female patients, and this is different from what was reported for HAT due to *T.b. gambiense* [11]. The reason for this scenario could not be ascertained in the current study. As reported in other studies [1, 12], HAT mainly affects people the most productive group (below 40 years), and this is different for malaria where children below 5 years are the most commonly affected.

The clinical signs observed in the current study are common in sleeping sickness patients and mainly include headache, sleeping disorders, fever, and general malaise amongst others [4, 9, 13]. Headache is an unspecific and common symptom amongst patients with HAT, and its frequency in the current study was higher than reported for *T.b. gambiense* patients in other countries (79%) [13]. Further, the prevalence of fever in the current study was higher than that reported in *T.b. gambiense* patients [13, 14]. This could be due to differences in the two trypanosome subspecies as well as higher prevalence of malaria in the current study. Sleeping disorders and other CNS manifestations were also common in the patients in the current study possibly because majority of them were in late-stage disease. The proportion of patients in the current study which had sleeping disorders was lower (45%) than that reported by Blum et al. [13] (74%) but was higher than those reported by Burri et al. [15] (29%) and Kuepfer et al. [4] (35%). In the late-stage HAT, the disease causes a dysregulation of the circadian rhythm of the sleep/wake cycle which is associated with insomnia and episodes of daytime sleepiness [13].

In spite of the importance of coinfections in management of tropical diseases, little is known about their occurrence. In most cases, HAT coinfections are only reported as case studies [16, 17] or as anecdotes [1, 13]. The current study shows that the commonest coinfections in patients with HAT include malaria and helminthosis. It is important to note that all HAT patients were also infected with malaria which reinforces the fact that the study area is highly endemic of malaria. In comparison, Blum et al. [13] reported a malaria prevalence of 50% in *T.b. gambiense* patients from several endemic countries. In Tanzania and Sudan, malaria was reported in 80% and 30% of the HAT patients, respectively [4, 12]. In western Kenya, malaria is caused by *P. falciparum* transmitted by culicine mosquitoes species that feed primarily on humans and cattle [18]. Sleeping sickness in the area is caused by *T.b. rhodesiense*, transmitted by *Glossina pallidipes *[19], and is zoonotic with animals such as cattle and pigs being the main reservoir of the disease [20]. Since the two diseases are coendemic, a combined control strategy may include use of insecticide-treated nets, clearance of the habitats harbouring the vectors, and spraying of livestock with acaricides. The strategies can employ existing synergy from the local institutions such as nongovernmental organizations (NGOs) and community-based organizations (CBOs) which have control programs on malaria and HIV [21].

Soil-transmitted parasites were also reported to be common amongst the HAT patients, with *A. duodenale*, *A. lumbricoides*, and *S. stercolaris* being the most commonly reported nematodes. A study in western Kenya by Brooker et al. [22] found that 91.7% of children were infected with similar range of helminths. Coinfections of *T.b. gambiense* and helminths have been reported in humans [12, 17]. Although the pathogenesis of trypanosomes and helminth coinfections have not been well studied in humans, animal model studies have shown that the interaction could be either agonistic and antagonistic depending on which of the parasites was inoculated first [23]. Infection with intestinal helminths was shown to be protective against malaria severity [24, 25], although this was disputed by other authors [26]. With their propensity for serial sampling, animal models can be utilized to determine the effects of concurrent infections on the host [23]. 

The occurrence of coinfections is bound to affect the susceptibility and pathogenesis of the diseases involved. In the current study, the effects of the coinfections on the immunology and hematology were not recorded, possibly due to the fact the hospital was only equipped for standard diagnosis of HAT. However, other studies have shown that HAT causes severe changes including immunosuppression and haematological disorders such as hemocytopenia and thrombocytopenia [1, 12, 27, 28]. Similar changes have been reported for malaria [29], and thus a patient having both infections might have poor prognosis. In case of further infection with immunosuppressive diseases such as HIV, the patient might have a fatal outcome, as what happened in the two cases reported in this study. HIV is highly endemic in the study area, where Busia District has the highest prevalence of HIV in Kenya in 2002 [16, 30]. In the current study, the patients were not routinely assessed for HIV, and thus it could not be ascertained whether the other patients had the disease. It would be important to determine the impact of HIV on the occurrence of other neglected diseases such as HAT. In recent years, HIV has been shown to increase the risk of patients having leishmaniasis by 100- to 1000-fold [27] while *T.b. gambiense* infection significantly lowers the sensitivity of HIV diagnostic tests [31]. 

At disease-management level, there is no clear guidance regarding the management of coinfections such as malaria and HAT. Thus, in HAT endemic areas the decision is left in the hands of the clinician regarding the mode of treatment [12]. In the current study, patients were treated with several drugs to clear the coinfections, and the toxicological implications of the consecutive use of these drugs on the patients are not known. When used alone, drugs such as suramin can cause complications while melarsoprol is considerably toxic [32]. As observed by Matete and Kajejo [16], the use of HAT drugs in patients having serious coinfections such as malaria and HIV is bound to increase their level of toxicities. For trypanostatic drugs such as eflornithine, a competent immune system is required to clear the infection, and it has been noted that eflornithine cannot cure HIV-positive, *T.b. gambiense* sleeping sickness patients [1, 33]. Further, melarsoprol was not curative in monkeys having a co-infection of *T.b. rhodesiense *and natural simian immunodeficiency virus (SIV) [34], and this could have been associated with immunosuppression caused by SIV. It would be important to determine the impact of using drug combinations for these coinfections in terms of mechanisms responsible for any toxicities and development of drug resistance.

## 5. Conclusion

In conclusion, the retrospective study showed that malaria and helminths are major coinfections found in patients having HAT in Kenya. Combined control strategies targeting these multiple diseases should be implemented. It would also be important to carry out further studies in patients and animal models to determine the impact of these coinfections on the immunopathogenesis and management of HAT.

## Figures and Tables

**Table 1:** Prevalence of coinfections in HAT patients (31) in NSSRH, Alupe, Kenya, from 2000 to 2009.

Disease	Number of patients	Prevalence (%) ± SE
Malaria	31	100
Helminthiasis	20	64.5 ± 8
*Ancylostoma duodenale *	12	38.7 ± 1
*Strongyloides stercolaris *	3	9.7 ± 5
*Ascaris lumbricoides *	14	45.2 ± 8
*Taenia* spp.	1	3.2 ± 3
*Trichomonas hominis*	7	22.6 ± 8
*Entamoeba *spp.	10	32.3 ± 9
HIV-AIDS	4	12.9 ± 4
Tuberculosis	1	3.2 ± 3
Typhoid	7	22.6 ± 8
Urinary tract infections	5	16.1 ± 6

**Table 2:** Clinical signs observed in patients at the point of admission at NSSRH, Alupe, Kenya, from 2000 to 2009.

Signs	Number of patients	Percentage (%)
Headache	23	74.2
Fever	15	48.4
*Sleep disorders (insomnia and daytime sleepiness)	14	45.2
General malaise	13	41.9
Joint pains	8	25.8
Abdominal distension	7	22.6
*Confusion	7	22.6
Pruritus	6	19.4
Nausea	6	19.4
Anorexia	6	19.4
Abdominal pain	6	19.4
Vomiting	5	16.1
General pain	5	16.1
Chest pain/congestion	5	16.1
Backache	4	12.9
*Stiff neck	4	12.9
*Coma	4	12.9
Coughing	4	12.9
Diarrhoea	4	12.9
Oedema of body parts	3	9.7
Breathing difficulties	3	9.7
*Lower limb weakness/numbness	3	9.7
*Restlessness	2	6.5
*Muscle pain	2	6.5
Sacral wounds	2	6.5
*Prostration	2	6.5
*Stupor	2	6.5
Palpitation	2	6.5
Incontinence	2	6.5
*Body chills	2	6.5
*Paralysis	2	6.5
*Dizziness	2	6.5
Dysuria	1	3.2
*Lower limb pain	1	3.2
Chancre	1	3.2
Dehydration	1	3.2
*Convulsions	1	3.2
*Reduced libido	1	3.2

*Late stage signs.

**Table 3:** Pattern of coinfections in 31 HAT patients in NSSRH, Alupe, Kenya, from 2000 to 2009.

Co-infection	Frequency	Percentage
HAT + malaria + helminth + GIT protozoa	9	0.29
HAT + malaria	8	0.26
HAT + malaria + helminth	4	0.13
HAT + malaria + helminth + GIT protozoa + UTI + typhoid	2	0.06
HAT + malaria + helminth + GIT protozoa + HIV	2	0.06
HAT + malaria + typhoid + UTI	2	0.06
HAT + malaria + TB + HIV	1	0.03
HAT + malaria + HIV + UTI + typhoid	1	0.03
HAT + malaria + helminth + typhoid	1	0.03
HAT + malaria + helminth + GIT protozoa + typhoid	1	0.03

HAT: human African trypanosomiasis, TB: tuberculosis, GIT: Gastro-intestinal, UTI: urinary tract infections, helminth: *Ancylostoma duodenale, Strongyloides stercolaris, Taenia *spp, protozoa: *Trichomonas hominis, Entamoeba *spp.
